# Health Care Comes Home: The Human Factors

**Published:** 2013-12-31

**Authors:** Stefanie Butz

**Affiliations:** Department of Primary Medical Care, University Medical Center Hamburg-Eppendorf, Germany, E-mail: s.butz@uke.de

This book is a result of a study assigned to the Committee on Human-Systems Integration of the National Research Council. The purpose was to conduct a widespread investigation of the role of human factors in home health care delivery trends and resulting challenges. To achieve that goal a 19-strong Committee was founded to explore home care issues through the lens of human factors and to make recommendations for improving the situation when health care is provided in the home environment.

The model of human factors is a user- or person-centred design, which by that is closely linked to the person-centred approach of integrated care.

The book contains seven chapters, the first one is an introduction to give an overview of the Committee and briefly explain the work phases, as well as a condensed view of the rise and the diversity of home health care in the USA.

Chapter 2 describes the current and future trends in population as well as detailed aspects of care recipients and caregivers.

The theory of human factors is explained in Chapter 3 using the model of human factors of health care in the home developed by Czaja and Nair. The model integrates the person(s) involved in health management, the tasks in which they are engaged, the equipment/technology that they are using to perform the task, and the environment in which these interactions occur. The goals of human factors are to optimize human- and system efficiency and effectiveness, safety, health, comfort and quality of life.

Chapter 4 shines a light on the health care tasks in home care. According to the authors one can categorize health care tasks into three main themes: (1) health maintenance, (2) care of injury, illness or impairments and (3) coordination of care. While health maintenance includes all tasks related to the activities of daily living and promoting general health and well-being as well as preventing diseases or disabilities, tasks under the second theme include episodic care, chronic care and end-of-life care. Finally, all tasks related to coordination and logistics of care, like scheduling medical appointments, arranging for transportation or renting or purchasing medical equipment, are summarised under the theme coordination of care.

The chapter also presents two methods to analyze health-related tasks in home care under the human factors approach and shows how to identify capabilities and information that are necessary to perform specific health-related tasks in a safe and effective way.

Chapter 5 handles health care technologies. According to the authors health care technologies can be subdivided into two main groups: (1) medical devices and (2) health information technologies. Whereas medical devices are clearly defined by the U.S. Food and Drug Administration and can be assigned into three classes, health information technologies are a growing field and no final definition or classification has been done. The authors then use different already existing devices and information technologies to explain the role of human factors in the design and evaluation of these technologies to prevent errors, or at least reduce the negative consequences of errors, and to better meet the needs of the users.

Chapter 6 deals with the home environment and states that the environment for home-based health care is multi-layered and includes factors of the physical, social, community and health policy environment. The authors discuss the impact of these factors on home care under the lens of human factors to explore how residents, health professionals and policy-makers can work together to end up with environments that support better health.

Finally chapter 7 presents conclusions and recommendations of the committee. The recommendations reflect the most critical steps that should be taken to improve health care at home and are separated into four categories: (1) health care technologies, (2) caregivers in the home, (3) residential environments for health care and (4) research and development.

The four recommendations under the category ‘health care technologies’ concern regulating technologies for health care consumers, developing guidance on the structure and usability of health information technologies, developing guidance and standards for medical device labeling and finally improving adverse event reporting systems for medical devices.

Under the category ‘Caregivers in the Home’ one recommendation is made that calls for more information and support for formal and informal caregivers.

Two recommendations are found under the category ‘Residential environments for health care’ and encourage modifications to existing housing as well as accessible and universal design for new housing.

Finally, the committee recommends: (1) research to enhance coordination among all the people who play a role in health care practice in the home, (2) development of a database of medical devices in order to facilitate device prescriptions, (3) improved surveys of the people involved in health care in the home and their residential environments and (4) development of tools for assessing the tasks associated with home-based health care.

In general, the book gives a good overview of health care at home in the USA. This is supported by the use of family vignettes in several chapters.

Yet, if one wants to use the book to get a deeper understanding and insight of the human factors approach it is not really suitable. The book only presents one model and the application of the approach on discussed topics is not always well explained. That would make it difficult if one tries to replicate the approach on other themes or topics.

In conclusion, this book can be recommended to people who are interested in applying the human factors approach and prefer an easy explanation. Further to people who are interested in policy-making and searching for an interesting approach to include people's capabilities and demands.

In the context of integrated care the book talks about a really important topic that not only the USA has to face in future. Every country has to find ways to take care of the elderly and the recommendation proposed by the committee could easily be transferred. Furthermore, the need for the use of new technologies will necessarily imply challenges and could be solved by integrated care.

